# Not All That Hurts Is Malignant: A Painful Case of Focal Nodular Hyperplasia

**DOI:** 10.7759/cureus.97990

**Published:** 2025-11-28

**Authors:** Zamanali Khakhar, Abdullahi Abdigafar Yunis, Dayib Buthul, Khadija Warfa, Soraiya Manji, Rajiv Patel, Sayed K Ali

**Affiliations:** 1 School of Medicine, University of Nairobi, Nairobi, KEN; 2 Department of Radiology, Aga Khan University Hospital, Nairobi, KEN; 3 Department of Obstetrics and Gynaecology, Aga Khan University Hospital, Nairobi, KEN; 4 Department of Internal Medicine, Aga Khan University Hospital, Nairobi, KEN

**Keywords:** benign hepatic lesion, central scar hepatic, fnh, focal nodular hyperplasia, hepatic mass

## Abstract

Focal nodular hyperplasia (FNH) is a benign hepatic lesion most often detected incidentally during imaging studies. While typically asymptomatic, a small subset of patients may present with abdominal pain or discomfort. Clinically and radiologically, FNH can mimic malignant liver tumors, occasionally resulting in unnecessary invasive procedures. This case report describes a 45-year-old woman with symptomatic FNH who was placed on routine imaging surveillance and managed conservatively. This case underscores the importance of accurately distinguishing this benign condition from hepatic malignancies. It further highlights the diagnostic challenges associated with achieving a definitive diagnosis.

## Introduction

Benign focal liver lesions are increasingly encountered due to the widespread use of abdominal imaging. They include a spectrum of non-malignant lesions, most commonly cavernous hemangiomas, followed by focal nodular hyperplasia (FNH) and hepatocellular adenomas, and are often discovered incidentally in patients with non-specific symptoms and without risk factors for liver malignancy [1]. Imaging modalities such as ultrasound, CT, and MRI play a pivotal role in detecting these lesions and distinguishing them from malignant tumors, thereby guiding appropriate clinical management.

FNH is a benign, tumor-like lesion of the liver and represents the second most common benign hepatic tumor after hemangioma [2]. Histologically, it is characterized by hyperplastic proliferation of hepatocytes, accompanied by the absence or malformation of normal portal tracts [3]. The challenge in differentiating FNH from other hepatic neoplasms, particularly hepatocellular adenomas, which often require surgical resection, remains a diagnostic challenge and a key clinical priority [4].

We present the case of a 45-year-old woman with symptomatic FNH, a condition that rarely manifests with clinical symptoms yet can closely mimic malignant hepatic processes. This case report underscores the importance of accurately distinguishing this benign lesion from other hepatic neoplasms and highlights the diagnostic challenges associated with its evaluation. Particular emphasis is placed on the role of various imaging and histopathological assessments in achieving diagnostic accuracy, as well as on contemporary management strategies and their alignment with current evidence.

## Case presentation

A 45-year-old Southeast Asian female schoolteacher presented to the outpatient clinic with acute-onset abdominal pain. The pain was located in the right upper quadrant and epigastric region, intermittent and dull in nature, and exacerbated by stretching or bending, but unrelated to meals. She denied associated symptoms such as nausea, vomiting, gastrointestinal bleeding, yellowing of the eyes or skin, or unintentional weight loss. Her medical and surgical histories were unremarkable. She did not consume alcohol or tobacco and reported an active lifestyle, averaging 7,000 steps per day. Family history was non-contributory.

On physical examination, the patient was hemodynamically stable with unremarkable vital signs. Mild tenderness was noted in the mid-epigastric region with no rebound tenderness, guarding, or organomegaly. Bowel sounds were present, and there were no disturbances in bowel or bladder habits. The remainder of the physical examination was unremarkable. Laboratory investigations were within normal limits (Table 1).

**Table 1:** Patient’s laboratory results. BUN = blood urea nitrogen; GGT = gamma-glutamyl transferase; AST = aspartate aminotransferase; ALT = alanine aminotransferase; ALP = alkaline phosphatase; PCR = polymerase chain reaction; AFP = alpha-fetoprotein

Test	Result	Reference range
BUN	5.54 mmol/L	3.20–8.20 mmol/L
Creatinine	74.21 µmol/L	62.00–115.00 µmol/L
GGT	21.30 U/L	0.00–38 U/L
AST	19.20 U/L	0.00–34 U/L
ALT	11.10 U/L	0.00–49 U/L
ALP	76.50 U/L	46–116 U/L
Serum lipase	68.80	0–160 U/L
HIV-1 RNA (PCR)	Negative	0–10,000,000 copies/mL
Hepatitis B surface antigen	Negative	
Hepatitis C antibody	Negative	
AFP	<21 ng/mL	<40 ng/mL
Serum β-hCG	<3 IU/L	<5 IU/L

**Figure 1:**
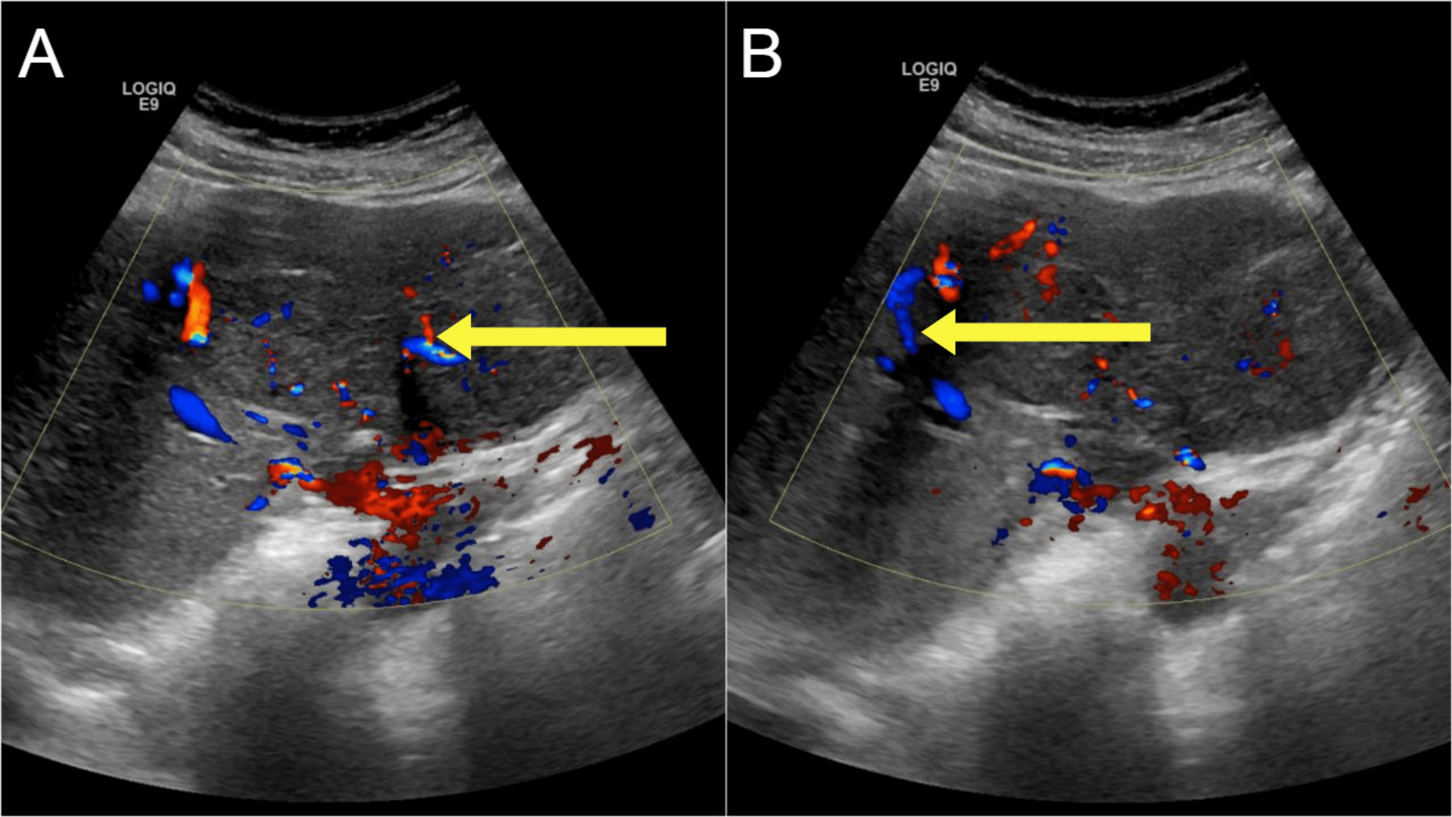
(A) Doppler ultrasound demonstrating the central vascular supply. (B) Doppler ultrasound demonstrating peripheral displacement of the vasculature.

**Figure 2:**
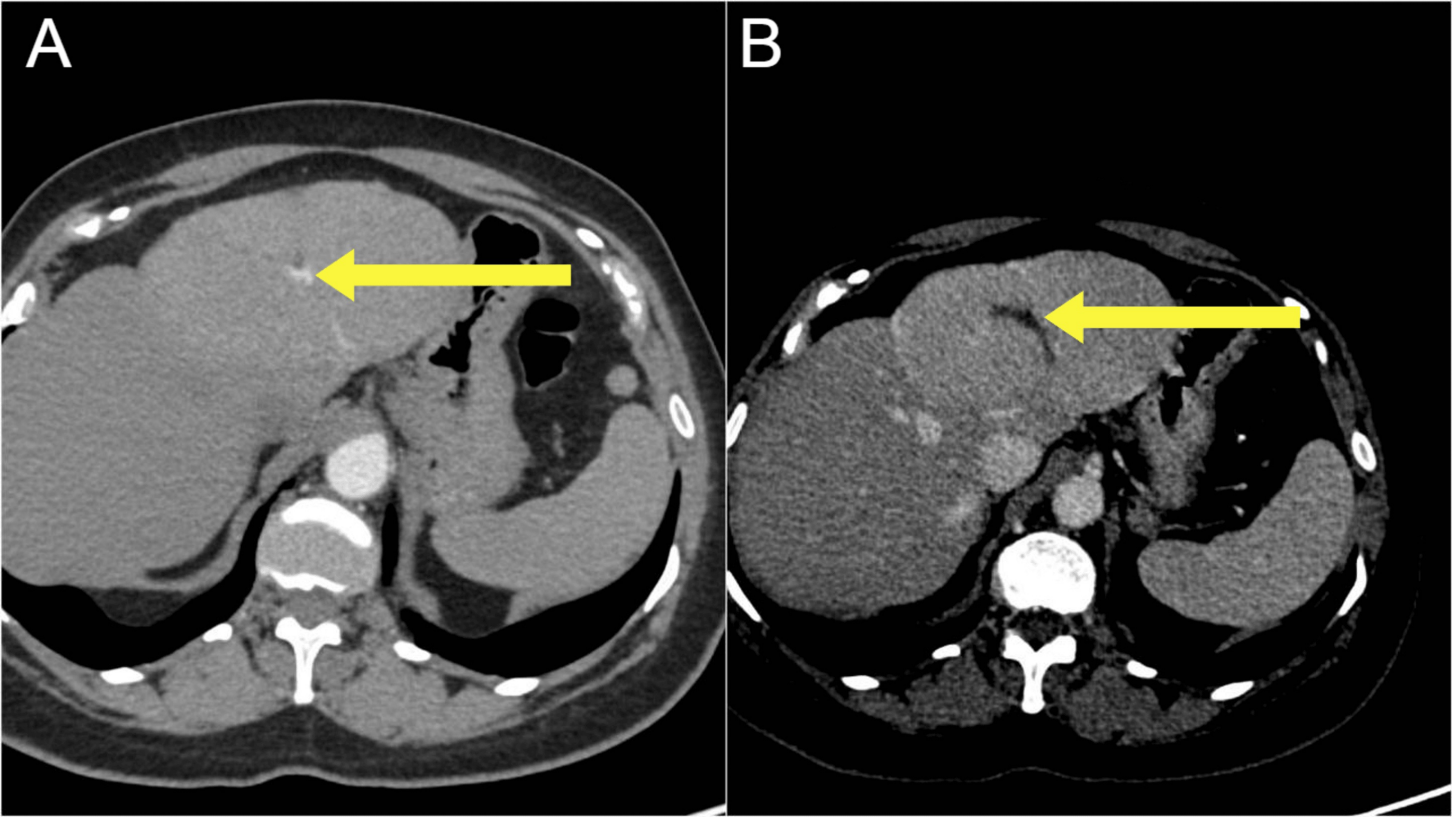
(A) Arterial-phase CT showing the feeding vessel in the center of the mass. (B) CT porto-venous liver window showing hyperattenuation of the mass compared to the normal liver parenchyma and the presence of a central scar.

**Figure 3:**
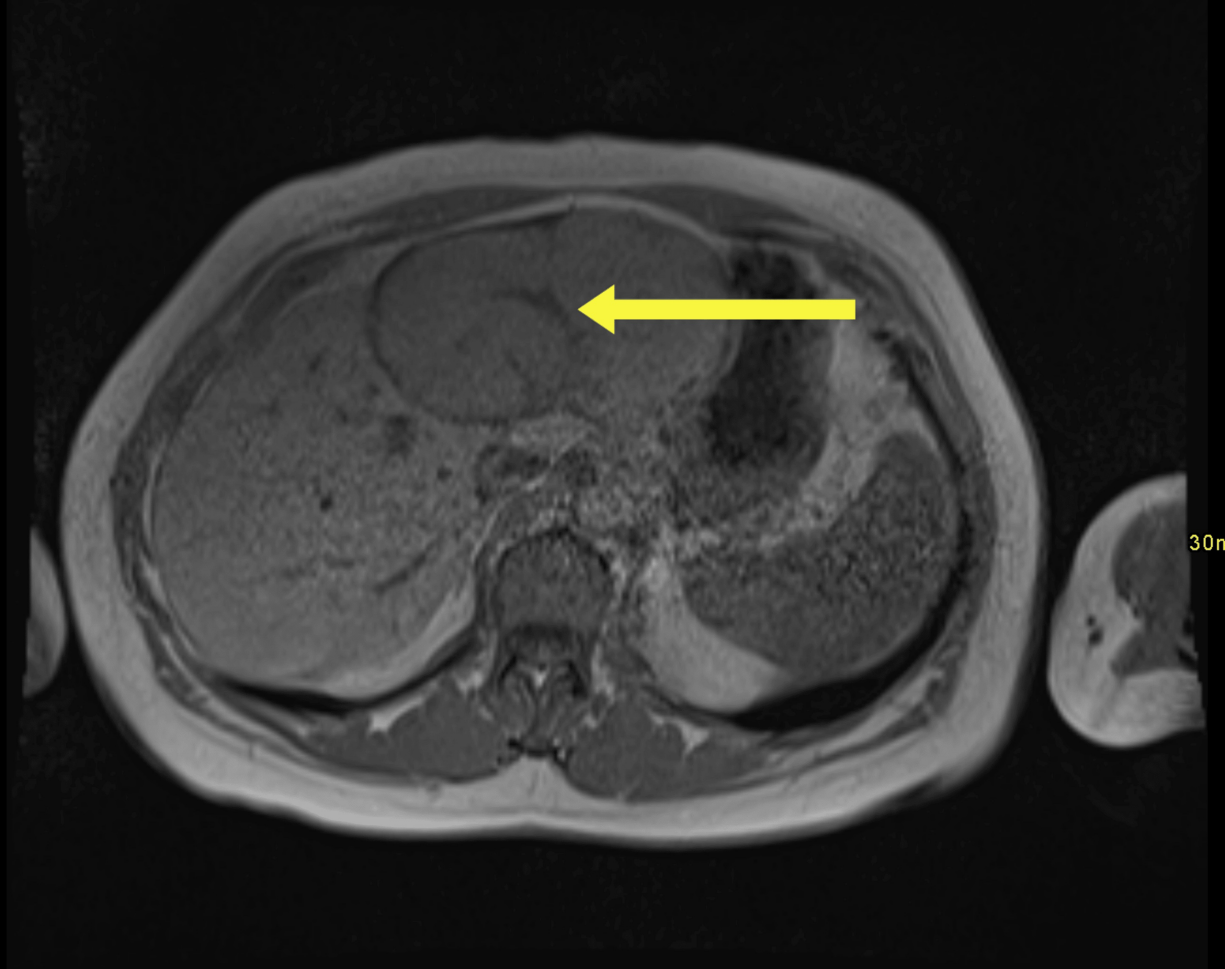
T1-weighted non-contrast image demonstrating a well circumscribed, isointense mass with a central hypointense stellate scar which is characteristic of focal nodular hyperplasia.

Abdominal ultrasound demonstrated a heterogeneous, ill-defined mass in the left hepatic lobe measuring approximately 10.1 × 6.3 cm. Doppler ultrasound revealed a central vascular supply characterized by a prominent feeding artery, along with peripheral displacement of the surrounding vasculature (Figure 1). A contrast-enhanced CT scan revealed a 7 × 11 × 8 cm mass in the same region, displaying enhancement characteristics consistent with FNH. Arterial-phase CT demonstrated a central feeding vessel within the mass, while the porto-venous phase showed hyperattenuation of the mass relative to the normal liver parenchyma, along with a distinct central scar (Figure 2). T1-weighted, non-contrast MRI demonstrated a well-circumscribed, isointense mass with a central hypointense stellate scar (Figure 3).

Following the characteristic radiologic findings confirming FNH, a biopsy was deemed unnecessary based on the high radiologic confidence in the diagnosis. The patient was managed conservatively with oral anti-inflammatories, given the benign nature of the mass and absence of concerning features. This led to the resolution of the abdominal pain. On follow-up, the pain was minimal and infrequent. She was scheduled for routine imaging surveillance at 6 and 12 months to monitor for stability in mass size and morphology. The patient was also counseled on recognizing symptoms such as sudden or severe abdominal pain and advised to seek prompt medical attention should these occur.

## Discussion

FNH represents a benign hyperplastic lesion of the liver, characterized by nodular masses of hepatic parenchyma organized around a central stellate scar, with associated abnormal vascular structures and radiating fibrous septa [5]. It has an incidence of 0.9%, with an overall prevalence in the general population ranging from 0.9% to 3%. FNH demonstrates a marked female predominance, yielding a female-to-male ratio of 8:1. The condition is identified in women between the third and fifth decades of life, and some studies suggest it is more common among premenopausal women. In contrast, male patients tend to be diagnosed at a significantly later age [3,6]. To date, no definitive racial predisposition has been established in the literature.

Due to its benign and typically asymptomatic nature, FNH is most often detected incidentally during radiological evaluation, usually as solitary lesions; however, multiple lesions may be observed in up to 20% of cases [5,7]. Although there is no consensus on the definitive etiology and pathogenesis of FNH, several postulations are agreed upon by researchers. The regenerative properties of the liver make it vulnerable to the development of atypical masses, which may be implicated in this condition [8]. Abnormal vascularization with arterial malformations within the liver alters perfusion, inducing a regenerative and hyperplastic response of otherwise normal hepatocytes [8,9]. It commonly presents as an isolated lesion but may also occur in association with other conditions such as hemangiomas, vasculitis, and hereditary hemorrhagic telangiectasia [8,10,11]. Some studies have demonstrated that prior administration of systemic chemotherapy agents may also play a role in the development of FNH [11,12].

FNH is asymptomatic in the majority of cases, but in patients with large nodules, it may manifest with non-specific abdominal pain secondary to pressure exerted on the capsule of the liver, as well as early satiety, dyspepsia, or symptoms due to mass effect on adjacent structures and a palpable, tender abdominal mass [6,8,13]. As it is known to be typically asymptomatic, the occurrence of painful or symptomatic lesions is rare [5]. It may progress to notable complications such as severe pain resulting from capsular distention in the presence of large nodules [13]. In a study conducted by Bonney et al., which followed 52 patients, a positive association was observed between nodule size and the occurrence of pain [14]. Spontaneous rupture of FNH with intralesional hemorrhage is an exceedingly rare complication, with approximately 10 cases described to date. Si et al. reported the case of a 43-year-old Chinese man with an 8.0 cm × 8.0 cm lesion who presented with spontaneous rupture and was managed surgically [10]. Mass effect and compression of adjacent structures can predispose to further complications [13].

Accurate diagnosis of FNH relies on triple-phase CT, MRI, and, occasionally, ultrasound-guided core biopsy, which overcomes the need for unnecessary invasive procedures [5,13]. The role of biopsy remains debated, but it may be warranted in rare instances, such as imaging uncertainty or when there is clinical suspicion of an underlying malignancy based on the physician’s judgement. Imaging typically demonstrates a central scar and abnormal vascular patterns. However, Hsee et al. reported the imaging findings of a patient in their study who lacked an obvious central scar, likely due to the lesion’s rapid growth and aggressive appearance [5]. Among the available modalities, MRI is both sensitive and specific, making it the most reliable for diagnosis. Contrast-enhanced ultrasound and Doppler studies may also provide valuable hemodynamic information regarding the vascular architecture [3,10]. Malignant neoplastic processes and other hypervascular lesions should be excluded to avoid misdiagnosis.

Asymptomatic patients with FNH are typically managed conservatively, with surgical resection reserved for those who develop symptoms or demonstrate rapid lesion growth. In the current case, due to the lack of rapid lesion growth, surgical resection was deferred in favor of conservative management, aligning with the patient’s preference. The decision was also guided by existing clinical guidelines and physician judgment at the time. Recent evidence suggests that routine follow-up imaging is unnecessary once the diagnosis of FNH has been firmly established, given its benign nature and minimal risk of malignant transformation [15]. Consistent with this, the European Association for the Study of the Liver guidelines recommend no further imaging surveillance when the diagnosis is confirmed radiologically [16]. However, in symptomatic patients, periodic imaging every 6-12 months may be appropriate to assess for changes in the size of the mass or characteristics. Surgical intervention should be considered only in cases of progressive enlargement, compressive symptoms, or increased risk of hemorrhage, particularly following trauma or in lesions associated with complications [13].

## Conclusions

FNH is a benign hepatic lesion that is most often asymptomatic and detected incidentally. Although symptomatic cases are rare, accurate diagnosis is critical, as FNH can closely mimic malignant liver tumors clinically. In this case, routine imaging surveillance and conservative management were the mainstay of care and allowed monitoring of lesion stability and provided symptomatic relief from abdominal pain. Advanced imaging remains the cornerstone for distinguishing FNH from other hepatic neoplasms, directly guiding appropriate management. This case underscores the importance of correlating imaging with clinical findings and recognizing the characteristic imaging features to ensure accurate diagnosis, prevent unnecessary invasive procedures, and avoid overtreatment.
